# Construction and validation of a predictive risk model for nosocomial infections with MDRO in NICUs: a multicenter observational study

**DOI:** 10.3389/fmed.2023.1193935

**Published:** 2023-06-26

**Authors:** Jinyan Zhou, Feixiang Luo, Jianfeng Liang, Xiaoying Cheng, Xiaofei Chen, Linyu Li, Shuohui Chen

**Affiliations:** ^1^Administration Department of Nosocomial Infection, Children’s Hospital, Zhejiang University School of Medicine, National Clinical Research Center for Child Health, Hangzhou, China; ^2^Neonatal Intensive Care Unit, Children's Hospital, Zhejiang University School of Medicine, National Clinical Research Center for Child Health, Hangzhou, China; ^3^Statistics Office, Children's Hospital, Zhejiang University School of Medicine, National Clinical Research Center for Child Health, Hangzhou, China; ^4^Quality Improvement Office, Children’s Hospital, Zhejiang University School of Medicine, National Clinical Research Center for Child Health, Hangzhou, China; ^5^Gastroenterology Department, Children's Hospital, Zhejiang University School of Medicine, National Clinical Research Center for Child Health, Hangzhou, China; ^6^Hangzhou Children's Hospital, Hangzhou, Zhejiang, China

**Keywords:** neonatal intensive care unit, multi-drug resistant organism, nosocomial infection, risk factor, predictive risk model

## Abstract

**Objectives:**

This study aimed to construct and validate a predictive risk model (PRM) for nosocomial infections with multi-drug resistant organism (MDRO) in neonatal intensive care units (NICUs), in order to provide a scientific and reliable prediction tool, and to provide reference for clinical prevention and control of MDRO infections in NICUs.

**Methods:**

This multicenter observational study was conducted at NICUs of two tertiary children’s hospitals in Hangzhou, Zhejiang Province. Using cluster sampling, eligible neonates admitted to NICUs of research hospitals from January 2018 to December 2020 (modeling group) or from July 2021 to June 2022 (validation group) were included in this study. Univariate analysis and binary logistic regression analysis were used to construct the PRM. H-L tests, calibration curves, ROC curves and decision curve analysis were used to validate the PRM.

**Results:**

Four hundred and thirty-five and one hundred fourteen neonates were enrolled in the modeling group and validation group, including 89 and 17 neonates infected with MDRO, respectively. Four independent risk factors were obtained and the PRM was constructed, namely: P = 1/ (1+ $e^{-X}$ ), *X* = −4.126 + 1.089× (low birth weight) +1.435× (maternal age ≥ 35 years) +1.498× (use of antibiotics >7 days) + 0.790× (MDRO colonization). A nomogram was drawn to visualize the PRM. Through internal and external validation, the PRM had good fitting degree, calibration, discrimination and certain clinical validity. The prediction accuracy of the PRM was 77.19%.

**Conclusion:**

Prevention and control strategies for each independent risk factor can be developed in NICUs. Moreover, clinical staff can use the PRM to early identification of neonates at high risk, and do targeted prevention to reduce MDRO infections in NICUs.

## Introduction

Neonates in neonatal intensive care units (NICUs), especially low-birth-weight and premature infants, are more likely to develop nosocomial infections due to various invasive procedures, long hospital stays, and weak innate immunity (1). The study of 29 European countries showed that the nosocomial infection rate of NICUs was 10.7%, which was significantly higher than that of neonatal wards (3.5%) (2). Nosocomial infections in neonates can prolong hospitalization, increase treatment costs, and even lead to death (3). In recent years, the problem of bacterial resistance has become increasingly serious and is spreading globally (4, 5). According to the World Health Organization (WHO) global surveillance report (6), the proportion of *Escherichia coli*, *Klebsiella pneumoniae*, and *Staphylococcus aureus* resistant to some commonly used antimicrobial drugs exceeded 50%. Multi-drug resistant organism (MDRO) mainly refers to the bacterium resistant to three or more antibiotics clinically (7), which has brought great difficulties to clinical treatment of infections and posed great threats to the prognosis of neonates. Nosocomial infections with MDRO accounted for more than 30% of all nosocomial infections in the NICU (8), and was significantly associated with higher neonatal mortality (9).

As an important part of etiology in epidemiology, predictive risk models (PRMs) can identify risk factors related to disease and determine the effect of each risk factor on disease by establishing a multi-factor statistical model, so as to identify risk groups and achieve early prevention and intervention. At present, PRMs for nosocomial infections with MDRO are mainly aimed at the patients after liver transplantation (10), patients with biliary tract infection (11), adult critical patients (12) and emergency patients (13), and there is no PRM for nosocomial infections with MDRO in NICUs.

Therefore, this study aimed to construct and validate the PRM for nosocomial infections with MDRO in NICUs, in order to provide a scientific and reliable prediction tool, and to provide reference for clinical prevention and control of MDRO infections in NICUs.

## Materials and methods

The research methods were determined in strict accordance with the ‘Transparent reporting of a multivariable prediction model for individual prognosis or diagnosis (TRIPOD): the TRIPOD statement’ (14).

### Study design, research setting

We conducted a multicenter observational study at NICUs of two tertiary children’s hospitals in Hangzhou, Zhejiang Province. The two NICUs have 30 and 50 beds respectively, mainly for the treatment of critical neonates with low birth weight, respiratory failure, complex congenital heart disease, severe brain injury, severe metabolic disorder, severe infection, and various congenital malformations. Since two research hospitals do not set up obstetrics, all neonates admitted to the two NICUs were born in other hospitals.

### Participants

Using cluster sampling, neonates who were admitted to NICUs of two research hospitals from January 2018 to December 2020 (modeling group) or from July 2021 to June 2022 (validation group), were diagnosed with nosocomial infections during hospitalization, and had complete medical records were included in this study. In particular, if neonates had more than one nosocomial infection during hospitalization, only the first nosocomial infection was analyzed.

### Data collection

In April 2021, 13 research variables including gestational age, birth weight, blood transfusion, duration of antibiotic use, breastfeeding, MDRO colonization, and maternal age were determined by systematic literature review and expert meeting method (see Supplementary material 1 for details and variable information). Modeling group data were collected retrospectively (January 2018 to December 2020) and validation group data were collected prospectively (July 2021 to June 2022). The data came from the critical care clinical information cloud system, hospital information system and hospital infection surveillance system. The data were entered independently by two people and reviewed by two others.

### Sample size

A total of 13 independent variables were included in this study. In the logistic regression, the recommended empirical criterion is that events per variable (EPV) should be at least 10 to ensure stable results (15). The study (8) concluded that the proportion of nosocomial infections with MDRO in NICU was 31.29%. Therefore, the sample size of the modeling group was at least 13 × 10÷31.29% = 415. A total of 435 neonates were included in the modeling group eventually.

The sample size of the validation group for external validation of the PRM was generally 1/4 ~ 1/2 of the sample size of the modeling group (16). So the sample size of the validation group was at least 435 × 1/4 = 109. A total of 114 neonates were included in the validation group actually.

### Outcome variables

Nosocomial infections with MDRO refer to nosocomial infections whose pathogens are MDROs. MDROs mainly refer to bacteria resistant to three or more antibiotics clinically (7), including vancomycin-resistant *Enterococcus* (VRE), carbapenems-resistant *Enterobacteriacea* (CRE), methicillin-resistant *Staphylococcus aureus* (MRSA), carbapenem-resistant *Pseudomonas aeruginosa* (CRPA), carbapenem-resistant *Acinetobacter baumannii* (CRAB), and extended-spectrum beta-lactamases (ESBLs) -producing *Enterobacteriaceae* (e.g., *Escherichia coli* and *Klebsiella pneumoniae*), etc. According to the <Diagnostic criteria for nosocomial infections (trial)> (17) issued by the Ministry of Health, China, nosocomial infections refer to infections acquired in the hospital by hospitalized patients, including infections occurred during hospitalization or acquired in the hospital and occurred after discharge (17). However, nosocomial infections do not include infections that occurred before admission or existed at admission (17).

### Statistical analysis

SPSS 26.0 (IBM Corporation) and R 4.2.1 (R Core Team) were used for data analysis.

#### Statistical description and univariate analysis

The categorical variables were compared using the Fisher’s exact test or *χ*^2^ test and expressed as percentages. The continuous variables were tested for normal distribution by the Kolmogorov–Smirnov test, and Mann–Whitney *U* test or One-Way ANOVA was used according to their distribution. Continuous variables of normal distribution were expressed by mean ± standard deviation, and continuous variables of non-normal distribution were expressed by median (interquartile range). *p* < 0.05 was considered statistically significant.

#### Multivariate analysis

Binary logistic regression was performed on variables that were statistically significant (*p* < 0.05) in univariate analysis to explore the independent risk factors for nosocomial infections with MDRO in NICUs. The binary logistic regression adopted likelihood ratio (LR: forward), the inclusion probability was 0.05, and the exclusion probability was 0.10.

#### Model construction

The regression equation was obtained according to the partial regression coefficient β of each independent risk factor and the constant term in the binary logistic regression results, and the PRM was constructed, namely P = 1/(1 + e^(−X)), X = β_0_ + β_1_X_1_ + β_2_X_2_ + β_3_X_3_ + … + β_m_X_m_. The R software was used to draw a nomogram based on the regression equation.

#### Model validation and evaluation

Hosmer-Lemeshow (H-L) test was used to evaluate the fitting degree of the model. *p* > 0.05 indicated that the PRM had a satisfactory fit (18). The calibration curve was used to reflect the calibration of the model. The discriminant validity of the model was usually tested by the area under the receiver operator characteristic curve (AUROC). It is generally believed that an AUROC value >0.70 indicates that the model has a relatively good discrimination (19). Decision curve analysis (DCA) was used to draw the DCA curves to evaluate the clinical effect of the model. The data of modeling group was resampled 1,000 times by Bootstrap method to validate the model internally. The data of the validation group were used to validate the model externally, and the accuracy of the model prediction was calculated.

### Ethics approval

The Ethics Committee of Children’s Hospital, Zhejiang University School of Medicine approved this study (2021-IRB-065). Informed consent has been obtained as required by the ethics committee.

## Results

### Basic characteristics of the research object

**Figure 1:**
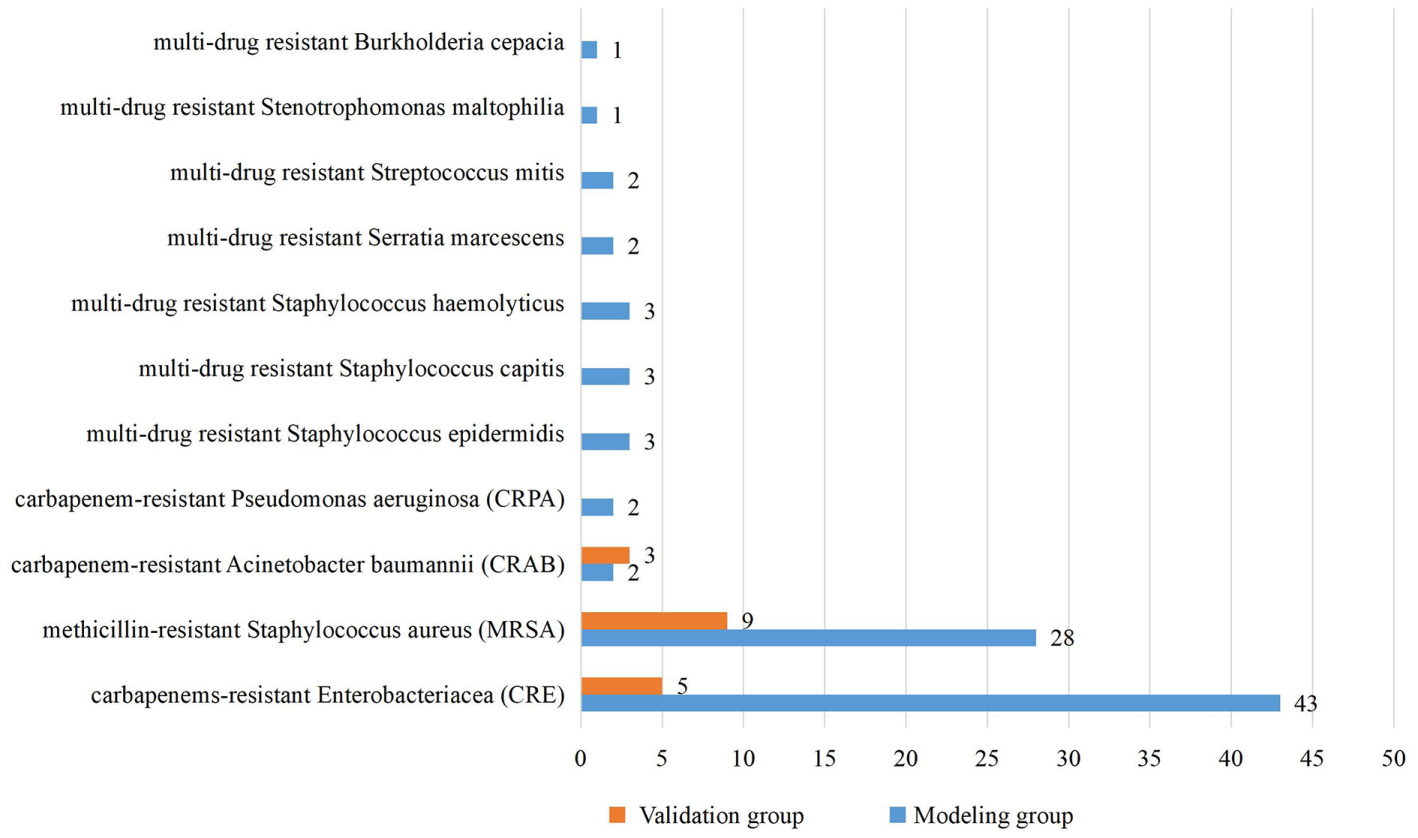
Pathogens of nosocomial infections with MDRO in NICUs.

From January 2018 to December 2020, a total of 4,727 neonates were admitted to the two NICUs of research hospitals, with 459 cases of nosocomial infection, the incidence rate of nosocomial infection was 9.71%, including 24 cases with incomplete medical records. Therefore, the modeling group included 435 neonates. From July 2021 to June 2022, a total of 1,301 neonates were admitted to the two NICUs of research hospitals, with 114 cases of nosocomial infection, the incidence rate of nosocomial infection was 8.76%. Due to complete medical records, 114 neonates were included in the validation group. Basic characteristics of the modeling group and the validation group are shown in Table 1. The modeling group and validation group had 261 and 64 males, and 89 and 17 neonates infected with MDRO, respectively. The median gestational age of both groups was 29 weeks, the median birth weights were 1,230 g and 1,280 g, and the median ages of admission were 2 and 3 days, respectively. There is no significant difference in basic characteristics between the two groups. The pathogens of MDRO infections in the modeling and validation groups were mainly CRE and MRSA (Figure 1).

**Table 1:** Basic characteristics of the modeling group and the validation group.

Characteristic		Modeling group (*n* = 435)	Validation group (*n* = 114)	*Z*/*χ*^2^	*p*
Gender, *n* (%)	Male	261 (60.00%)	64 (56.14%)	0.56	0.46
	Female	174 (40.00%)	50 (43.86%)		
Gestational age (weeks), Median (IQR)		29 (27, 32)	29 (27, 33)	1.25	0.21
Birth weight (g), Median (IQR)		1,230 (980, 1,640)	1,280 (943.75, 2351.25)	−0.29	0.77
With infectious diseases at admission, *n* (%)	Yes	100 (22.99%)	33 (28.95%)	1.75	0.19
	No	335 (77.01%)	81 (71.05%)		
Age of admission (days) Median (IQR)		2 (1，13)	3 (1，17.25)	1.01	0.31
Nosocomial infection with MDRO, *n* (%)	Yes	89 (20.46%)	17 (14.91%)	1.78	0.18
	No	346 (79.54%)	97 (85.09%)		

IQR, interquartile range, MDRO: multi-drug resistant organism.

### Univariate analysis

The neonates in the modeling group were divided into the MDRO infection group and the non-MDRO infection group according to whether the pathogen was a MDRO. Univariate analysis of 13 variables was conducted to initially explore the differences between the two groups (Table 2). As can be seen from the table, there were 8 variables with statistically significant differences between the two groups. Compared with the non-MDRO infection group, the MDRO infection group was more likely to have the following characteristics: premature, low birth weight, maternal age ≥ 35 years, blood transfusion, blood collection >10 times, use of antibiotics >7 days, MDRO colonization and length of stay >10 days (*p* < 0.05).

**Table 2:** Univariate analysis of the modeling group (*n* = 435).

Variable	MDRO infection group (*n* = 89)	non-MDRO infection group (*n* = 346)	*Z*/*χ*^2^	*p*
Premature^a^, *n* (%)	85 (95.51%)	284(82.08%)	9.91	<0.01
Low birth weight^b^, *n* (%)	85 (95.51%)	284(82.08%)	9.91	<0.01
With infectious diseases at admission, *n* (%)	25 (28.09%)	75 (21.68%)	1.65	0.20
Maternal age ≥ 35 years^c^, *n* (%)	40 (44.94%)	54 (15.61%)	35.97	<0.01
Breastfeeding, *n* (%)	63 (70.79%)	230 (66.47%)	0.60	0.44
Invasive mechanical ventilation, *n* (%)	65 (73.03%)	227 (65.61%)	1.77	0.18
Blood transfusion, *n* (%)	78 (87.64%)	246 (71.10%)	10.19	<0.01
Use of vascular catheter for ≥7 days^d^, *n* (%)	50 (56.18%)	179 (51.73%)	0.56	0.45
Blood collection >10 times^e^, *n* (%)	49 (55.06%)	134 (38.73%)	7.74	<0.01
Use of antibiotics >7 days^f^, *n* (%)	72 (80.90%)	169 (48.84%)	29.44	<0.01
Combination of antibiotics, *n* (%)	56 (62.92%)	179 (51.73%)	3.57	0.06
MDRO colonization, *n* (%)	49 (55.06%)	138 (39.88%)	6.65	0.01
Length of stay >10 days^g^, *n* (%)	81 (91.01%)	257 (74.28%)	11.44	<0.01

MDRO, multi-drug resistant organism.

a Preterm infants were defined as those born at a gestational age of < 37 weeks (≤259 days).

b Low birth weight infants were defined as those whose birth weight was < 2,500 g.

c Advanced maternal age is generally considered as maternal age ≥ 35 years, so the cut-off value was determined by this.

d, f The cut-off values were determined according to the relevant research results.

e, g The cut-off values were determined according to the ROC curves.

### Multivariate analysis

Collinearity diagnosis was performed on the above 8 variables that were significant in univariate analysis. The results showed that the tolerances of 8 variables were all >0.10, the variance inflation factors (VIFs) of 8 variables were all <10.0 (Specific results are presented in Supplementary material 2). It can be considered that there is no multicollinearity among the 8 variables (20), and logistic regression can be carried out. Binary logistic regression revealed that the independent risk factors of nosocomial infections with MDRO in NICUs were: low birth weight (OR: 2.97, 95%CI: 1.00 ~ 8.82, *p* < 0.05), maternal age ≥ 35 years (OR: 4.20, 95%CI: 2.43 ~ 7.26, *p* < 0.01), use of antibiotics >7 days (OR: 4.47, 95%CI: 2.45 ~ 8.17, *p* < 0.01) and MDRO colonization (OR: 2.20, 95%CI: 1.31 ~ 3.71, *p* < 0.01). The specific results of binary logistic regression are shown in Supplementary material 3.

### PRM construction

According to the partial regression coefficient β of each independent risk factor and constant term in the logistic regression results, the logistic regression equation was obtained and the PRM was constructed, namely:

P = 1/(1+ $e^{-X}$ ), *X* = −4.126 + 1.089× (low birth weight) +1.435× (maternal age ≥ 35 years) +1.498× (use of antibiotics >7 days) + 0.790× (MDRO colonization).

**Figure 2:**
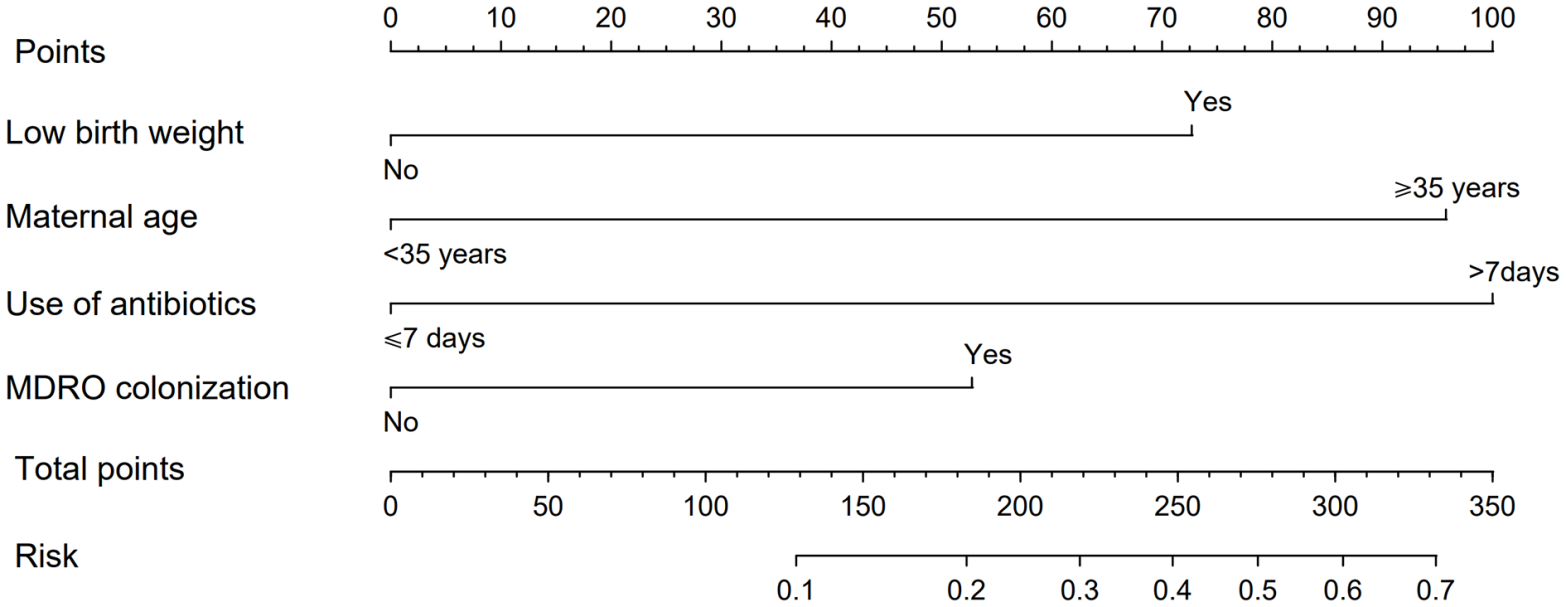
Nomogram of PRM for nosocomial infections with MDRO in NICUs. The method of using the nomogram was as follows: according to the actual situation of each risk factor in the neonate, the corresponding risk point was obtained through the vertical line (corresponding to the top of the nomogram), the risk point of each risk factor was summed to obtain the total risk points (corresponding to the total points line of the nomogram), and the risk value corresponding to the total points was obtained through the vertical line (corresponding to the bottom of the nomogram).

In order to make the PRM more intuitive and more convenient for clinical application, the above logistic regression equation was drawn into a nomogram to visualize the PRM (Figure 2). The nomogram is used as follows. According to the actual situation of each risk factor of the neonate, the corresponding risk points (corresponding to the ‘Points’ line at the top of the nomogram) can be obtained by drawing a vertical line. Add up the risk points of each risk factor to get the total risk points (corresponding to the “Total points” line of the nomogram). Finally, the risk value (corresponding to the “Risk” line at the bottom of the nomograph) corresponding to the total points can be obtained by drawing a vertical line.

### PRM validation

**Figure 3:**
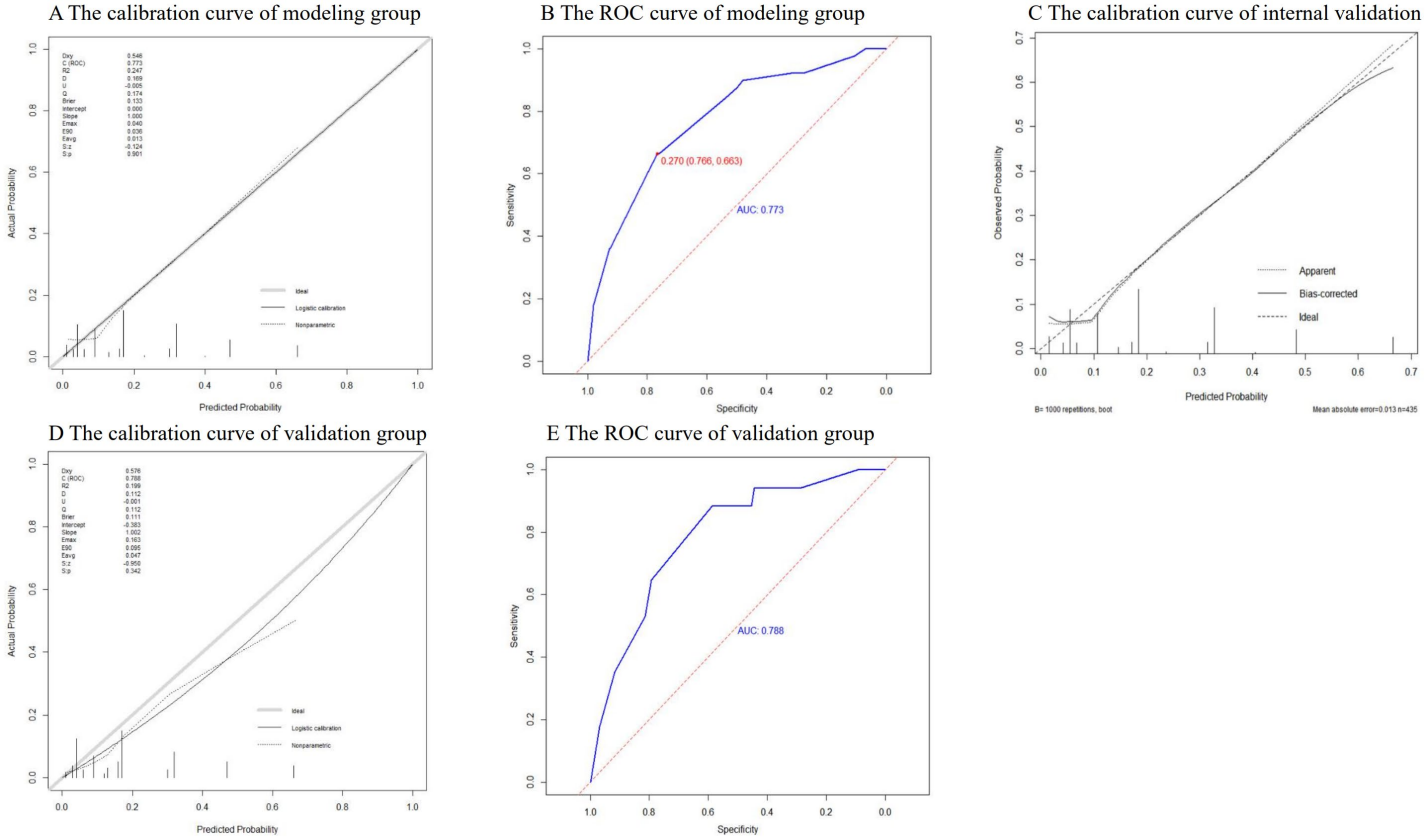
The results of internal and external validation.

H-L test was conducted on the model in the modeling group and the validation group, and the *p* values were 0.61 and 0.49 (>0.05), respectively. It can be considered that there was no significant difference between the model predicted value and the actual value, and the model fit was good. The calibration curves were close to *y* = *x*, indicating that the predicted probability of the model was in good agreement with the actual probability (Figures 3A,D). The AUROC values of the modeling group and the validation group were 0.773 (95% CI: 0.718 ~ 0.828) and 0.788 (95% CI: 0.677 ~ 0.899) respectively, indicating that the model had a relatively good discrimination (Figures 3B,E). Using Bootstrap method, the data of modeling group was repeatedly sampled 1,000 times to calculate the corrected C-index, and the result was 0.766, indicating that the model was stable and the discrimination was good. The corresponding calibration curve was drawn, which was close to *y* = *x*, indicating that the predicted value fitted in well with the observed value (Figure 3C).

### The prediction effect of the PRM

The ROC curve of the modeling group showed that the optimal cut-off value was when the predicted risk value was 0.27 (Figure 3B). Based on this, risk stratification was performed, that is, when the predicted probability was ≥0.27, the risk of nosocomial infections with MDRO was high, and when the predicted probability is <0.27, the risk of nosocomial infections with MDRO was low. According to the PRM, the prediction probability of nosocomial infections with MDRO of each neonate in the validation group was calculated, and the prediction effect of the model was tested. The results showed that in the validation group, the PRM predicted that 11 out of 17 cases of MDRO infection group were at high risk, and 77 out of 97 cases of non-MDRO infection group were at low risk. Compared with the actual results, the sensitivity of the prediction results was 64.71%, the specificity was 79.38%, the Jordan index was 0.44, the positive likelihood ratio was 3.14, the negative likelihood ratio was 0.44, and the prediction accuracy was 77.19%.

### The clinical validity of the PRM

**Figure 4:**
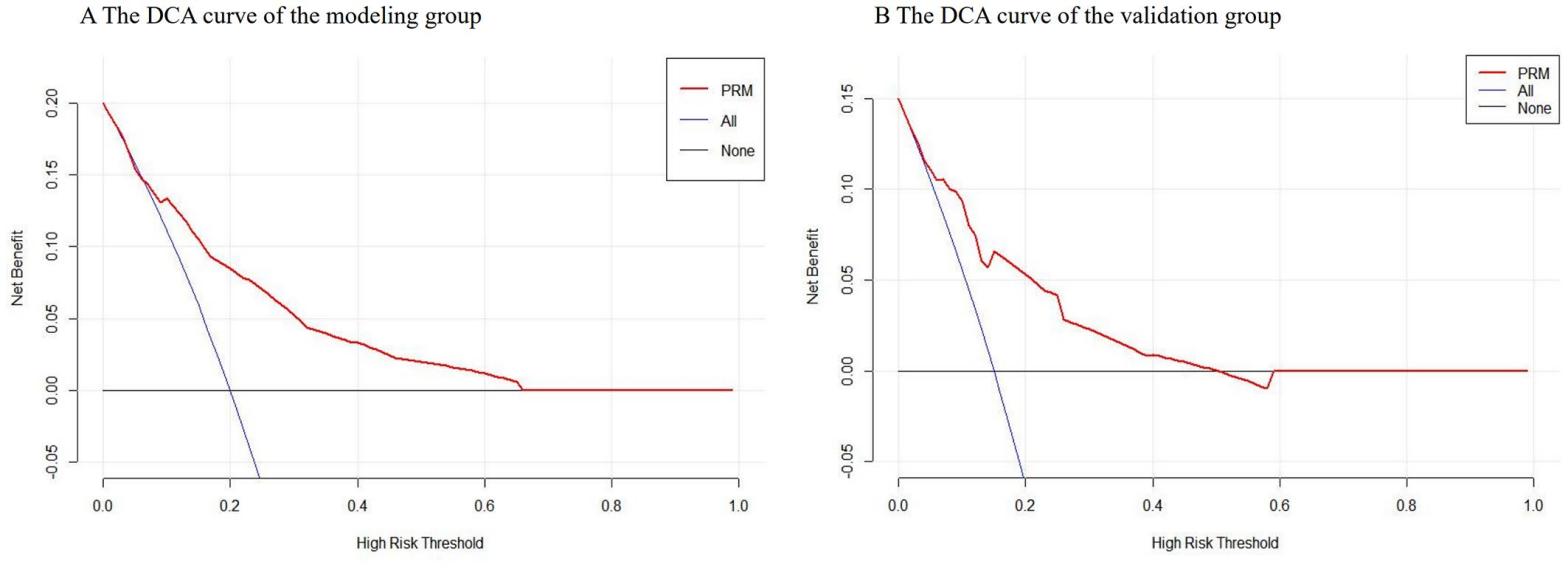
The DCA curves of the modeling group and the validation group.

Decision curve analysis (DCA) was used to calculate the net benefit at each threshold probability in this study. The DCA curves of the modeling group and the validation group are shown in Figures 4A,B respectively. Suppose the PRM predicts that the probability of nosocomial infections with MDRO in neonate *i* is Pi. When Pi reaches a certain threshold (noted as Pt), it is considered that neonate *i* will have nosocomial infections with MDRO, and intervention measures will be taken. The abscissa is the threshold probability, and the ordinate is the net benefit of taking interventions. The black horizontal line and the blue slash line represented two extreme cases. The black horizontal line indicated that none of the neonates experienced nosocomial infections with MDRO and none received interventions, at which point the net benefit was zero. The blue slash line showed the net benefit when all neonates developed nosocomial infections with MDRO and all received interventions. The red curve represented the net benefit of performing interventions based on this study’s PRM. The results showed that the red line was located above the black and blue lines when the threshold probabilities were 7% ~ 65 and 3% ~ 50% in the modeling group and the validation group, respectively. At this time, interventions based on the PRM can achieve greater net benefits, indicating that the PRM constructed in this study had certain clinical validity.

## Discussion

With the rapid development of the Internet and the in-depth integration with the medical and health industry, relying on artificial intelligence and machine learning technology, the treatment, nursing and health management of patients gradually tend to be individualized and refined (21, 22). In this study, 13 research variables were identified through systematic literature review and expert meeting method. After univariate analysis and binary logistic regression analysis, 4 independent risk factors were attained. The PRM for nosocomial infections with MDRO in NICUs was constructed and a nomogram was drawn. The internal and external validation showed that the PRM had good calibration and discrimination, relatively accurate prediction, and had certain clinical validity, which can provide scientific basis for prevention and control of nosocomial infections with MDRO in NICUs.

The 4 independent risk factors obtained in this study were low birth weight, maternal age ≥ 35 years, use of antibiotics >7 days and MDRO colonization. Therefore, to reduce MDRO infections in NICUs, care for low birth weight infants should be enhanced. Appropriate marriage and childbearing should be advocated, and pregnant women, especially those with advanced maternal age, should take good prenatal care. Nursing staff should strengthen the postpartum care for elderly puerperae and closely observe their neonates. Rational use of antibiotics is particularly important to prevent MDRO infections. Doctors should administer antibiotics in strict accordance with the indications for the application of antibiotics, and nurses should feedback the effect of medication in time and cooperate with doctors to use drugs rationally. In addition, intensive MDRO surveillance of neonates should be undertaken in ways such as active screening, and prompt decolonization therapy should be offered to neonates with MDRO colonization.

In addition to guiding NICUs to carry out prevention and control strategies for each independent risk factor, based on the results of this study, clinical staff can use the PRM to identify high-risk neonates at an early stage, and give special attention to improve the effect of infection prevention and control, reduce the incidence of infection, and ultimately improve the treatment effect and prognosis of neonates.

With the continuous development of global bacterial resistance, the situation of nosocomial infections with MDRO is becoming more and more serious. Nosocomial infections with MDRO greatly increase the difficulty of treatment, increase the financial burden on patients’ families, and affect patient prognosis, even leading to patient death. In this context, constructing a PRM will help identify susceptible populations, achieve early prevention and intervention, so as to improve patient prognosis and medical quality. As far as we know, this study is the first to construct a PRM for nosocomial infections with MDRO in NICUs. This study provides a relatively scientific and relatively reliable prediction tool, and can provide reference for clinical prevention and control of MDRO infections in NICUs. However, there are some limitations to this study. Firstly, a retrospective design was adopted in the construction of the PRM, which may affect the accuracy of the results due to information bias. Secondly, since only two hospitals were selected, the study subjects were not sufficiently represented, and the generalizability of the findings needs to be further verified. Moreover, the external validation in this study selected research subjects from the same institutions in different periods rather than from different institutions, which makes it impossible to determine whether the model constructed in this study is applicable to other regions or other institutions. Finally, because the study sites of this study were tertiary children’s hospitals, the applicability of the PRM to NICUs of other types of hospitals remains to be tested.

## Conclusion

Our study constructed a PRM for nosocomial infections with MDRO in NICUs. After validating, the PRM had a good calibration degree and discriminant validity, relatively accurate prediction, and had some clinical application value. Based on the findings of this study, prevention and control strategies for each independent risk factor can be developed in NICUs. At the same time, clinical staff can take advantage of the PRM to enable early identification of neonates at high risk of MDRO infections, and thus do targeted prevention to reduce the occurrence of nosocomial infections with MDRO in NICUs.

## Data availability statement

The original contributions presented in the study are included in the article/Supplementary material, further inquiries can be directed to the corresponding author.

## Ethics statement

The studies involving human participants were reviewed and approved by the Ethics Committee of Children’s Hospital, Zhejiang University School of Medicine (2021-IRB-065). Informed consent has been obtained as required by the ethics committee.

## Author contributions

JZ conceptualized and designed the study, collected data, analyzed data, drafted the initial manuscript, and critically reviewed and revised the manuscript. FL conceptualized and designed the study, analyzed data, and critically reviewed and revised the manuscript. JL directed and supervised data analysis and critically reviewed and revised the manuscript. XYC reviewed data collection, and critically reviewed and revised the manuscript. XFC reviewed data collection, and critically reviewed and revised the manuscript. LL collected data and critically reviewed and revised the manuscript. SC conceptualized and designed the study, coordinated and supervised data collection and analysis, and critically reviewed and revised the manuscript for important intellectual content. All authors approved the final manuscript as submitted and agree to be accountable for all aspects of the work.

## Conflict of interest

The authors declare that the research was conducted in the absence of any commercial or financial relationships that could be construed as a potential conflict of interest.

## Publisher’s note

All claims expressed in this article are solely those of the authors and do not necessarily represent those of their affiliated organizations, or those of the publisher, the editors and the reviewers. Any product that may be evaluated in this article, or claim that may be made by its manufacturer, is not guaranteed or endorsed by the publisher.
