# Supplementary material for: Construction and validation of a predictive risk model for nosocomial infections with MDRO in NICUs: a multicenter observational study
Source: Front Med (Lausanne). 2023 Jun 26;10:1193935. doi: 10.3389/fmed.2023.1193935 (PMC10332151; doi:10.3389/fmed.2023.1193935)
Supplement: Supplementary file 1 [file Table_1.DOCX]

**Supplementary Material 1**

A total of 10 literatures ^[1-10]^ were included through systematic literature review. 17 influencing factors were summarized from the included literatures. Based on expert opinions, among the 17 influencing factors, 8 variables were included, 6 variables were deleted, 3 variables were modified to 2 new variables, and 3 new variables were added. Finally, 13 research variables were obtained. The determination process of research variables is shown in the following table.

| Category | Factors | Included/Deleted/Modified/Added |
| --- | --- | --- |
| Neonate | gestational age | Included |
|  | birth weight | Included |
|  | first birth | Deleted  Reason: Although the previous study concluded that ‘first birth’ and ‘in vitro fertilization’ were risk factors, by retrospecting the relevant literature ^[10]^, it was found that the results were only based on univariate analysis rather than multivariate analysis, so the reference value was low. Considering that these two variables had little clinical significance, it was recommended to delete them. |
|  | in vitro fertilization |  |
|  | coma | Deleted  Reason: The neonates in NICUs are often in critical condition, requiring intubation and sedative drugs. It is difficult to identify whether the neonate has coma accurately. |
|  | with kidney disease | Deleted  Reason: According to the clinical work experience and the confirmation of the medical record system, the number of neonates with kidney disease admitted by NICUs is very small. |
|  | eyes secreting mucus | Modified to ‘with infectious diseases at admission’  Reason: ‘Eyes secreting mucus’ indicates that the neonate may have infection in eyes, and it was recommended to change it to ‘with infectious diseases at admission’, including congenital infection, sepsis/bacteremia, meningitis, infectious pneumonia, conjunctivitis, etc., to detect the impact of the status of the neonate at admission. |
| Maternal | age | Added  Reason: According to the clinical work experience, the neonates born to advanced maternal age (≥35 years old) are more likely to get sick, and the advanced maternal age may be related to neonatal infections, so it is suggested to include the maternal age as a variable. |
| Treatment | invasive mechanical ventilation | Included |
|  | Umbilical Venous Catheters | Modified to ‘vessel catheter’  Reason: Common vascular catheters in clinical practice include Peripherally Inserted Central Catheter (PICC), Central Venous Catheter (CVC), Umbilical Venous Catheters (UVC), Umbilical Artery Catheters, UAC), Peripheral Venous Catheter (PVC), peripheral arterial catheter, etc.. |
|  | Peripherally Inserted Central Catheter |  |
|  | combined use of antibiotics | Included |
|  | duration of antibiotic use | Included |
|  | number of days from admission to isolation | Deleted  Reason: NICU doctors issue isolation orders for neonates diagnosed with nosocomial infections. Therefore, ‘number of days from admission to isolation’ is the same as ‘length of stay’ when a neonate has nosocomial infection, and it was recommended to delete it. |
|  | breast feeding | Included |
|  | kangaroo mother care | Deleted  Reason: Affected by the COVID-19 pandemic, the ‘kangaroo mother care’ has been cancelled in NICUs. |
|  | MDRO colonization | Included |
|  | length of stay | Included |
|  | transfusion | Added  Reason: The incidence of blood related infection is high in clinic.  The incidence of blood-related infections is relatively high in clinical practice. According to clinical work experience, it was suggested to add two variables of ‘blood transfusion’ and ‘blood collection’ so as to more fully examine the impact of vascular invasive procedures. |
|  | blood collection |  |

[1] Xu L, Wang RY, Chen BB, et al. Risk factors and prevention measures of multiple drug-resistant infections in neonatal intensive care unit. *Chin J General Pract*. (2018) 16:1314−1317.

[2] Tsai MH, Chu SM, Hsu JF, et al. Risk factors and outcomes for multidrug-resistant Gram-negative bacteremia in the NICU. *Pediatrics*. 2014;133(2):e322-e329.

[3] Xie ZY, Xiong Y, Sun J, et al. Logistic regression analysis of risk factors of multiple drug-resistant infections in neonatal intensive care unit. *Journal of Chinical Pediatrics*. 2016,34(09):641-644.

[4] Zhang L, Xie F. The distribution，drug susceptibility of pathogenic bacteria infections in neonatal intensive care unit and regression analysis for the related risk factors. *Hebei Medical Journal*. 2018,40(05):775-777.

[5] Wu JC, Yang JQ, Liang D, et al. Distribution of multidrug-resistant organism infections in neonates of NICU and risk factors [J]. *Chinese Journal of Nosocomiology*. 2016,26(01):191-194.

[6] Sultan AM, Seliem WA. Identifying Risk Factors for Healthcare-Associated Infections Caused by Carbapenem-Resistant *Acinetobacter baumannii* in a Neonatal Intensive Care Unit. *Sultan Qaboos Univ Med J*. 2018;18(1):e75-e80.

[7] Kumar A, Randhawa VS, Nirupam N, et al. Risk factors for carbapenem-resistant Acinetobacter baumanii blood stream infections in a neonatal intensive care unit, Delhi, India. *J Infect Dev Ctries*. 2014;8(8):1049-1054.

[8] Sakaki H, Nishioka M, Kanda K, et al. An investigation of the risk factors for infection with methicillin-resistant Staphylococcus aureus among patients in a neonatal intensive care unit. *Am J Infect Control*. 2009;37(7):580-586.

[9] Maraqa NF, Aigbivbalu L, Masnita-Iusan C, et al. Prevalence of and risk factors for methicillin-resistant Staphylococcus aureus colonization and infection among infants at a level III neonatal intensive care unit. *Am J Infect Control*. 2011;39(1):35-41.

[10] Hadžić D, Skokić F, Brkić S, et al. Epidemiology of neonatal sepsis caused by multidrug resistant pathogens in a neonatal intensive care unit level 3. *Med Glas (Zenica)*. 2020;17(2):375-382.

The research variables of this study are shown in the table below. It should be noted that since the purpose of this study was to analyze the risk factors and to construct a predictive risk model, the variable information in the ‘Treatment’ section should record the time between admission to the NICUs and the diagnosis of nosocomial infections.

| Category | Variables |
| --- | --- |
| Neonate | gestational age, birth weight, with infectious diseases ^a^ at admission |
| Maternal | age |
| Treatment | invasive mechanical ventilation, vessel catheter ^b^, blood transfusion, blood collection, combination of antibiotics ^c^, duration of antibiotic use, breastfeeding, MDRO colonization ^d^, length of stay |

^a^ Including congenital infection, sepsis/bacteremia, meningitis, infectious pneumonia, conjunctivitis, etc.

^b^ Including Peripherally Inserted Central Catheter (PICC), Central Venous Catheter (CVC), Umbilical Venous Catheters (UVC), Umbilical Artery Catheters, UAC), Peripheral Venous Catheter (PVC), peripheral arterial catheter, etc.

^c^ It refers to the use of two or more antibacterial drugs at the same time.

^d^ It refers to the detection of MDRO in neonatal specimens, but the neonate has not yet experienced symptoms of nosocomial infections.
