# Supplementary material for: Construction and validation of a predictive risk model for nosocomial infections with MDRO in NICUs: a multicenter observational study
Source: Front Med (Lausanne). 2023 Jun 26;10:1193935. doi: 10.3389/fmed.2023.1193935 (PMC10332151; doi:10.3389/fmed.2023.1193935)
Supplement: Supplementary file 2 [file Table_2.DOCX]

**Supplementary Material 2**

The results of collinearity diagnosis

| Variable | Tolerance | Variance Inflation Factor (VIF) |
| --- | --- | --- |
| premature | 0.29 | 3.45 |
| low birth weight | 0.29 | 3.42 |
| maternal age ≥35 years | 0.97 | 1.03 |
| blood transfusion | 0.80 | 1.26 |
| blood collection >10 times | 0.70 | 1.42 |
| use of antibiotics >7 days | 0.84 | 1.19 |
| MDRO colonization | 0.96 | 1.04 |
| length of stay >10 days | 0.73 | 1.38 |
