# Supplementary material for: Construction and validation of a predictive risk model for nosocomial infections with MDRO in NICUs: a multicenter observational study
Source: Front Med (Lausanne). 2023 Jun 26;10:1193935. doi: 10.3389/fmed.2023.1193935 (PMC10332151; doi:10.3389/fmed.2023.1193935)
Supplement: Supplementary file 3 [file Table_3.DOCX]

**Supplementary Material 3**

The results of binary logistic regression

| Risk factor | β | SE | OR (95%CI) | *P* |
| --- | --- | --- | --- | --- |
| Low birth weight | 1.089 | 0.56 | 2.97（1.00，8.82） | 0.0498 |
| Maternal age ≥35 years | 1.435 | 0.28 | 4.20（2.43，7.26） | <0.01 |
| Use of antibiotics >7 days | 1.498 | 0.31 | 4.47（2.45，8.17） | <0.01 |
| MDRO colonization | 0.790 | 0.27 | 2.20（1.31，3.71） | <0.01 |
| Constant term | -4.126 | 0.61 | 0.02 | <0.01 |

SE: standard error, OR: odds ratio, CI: confidence interval
